# An Overlooked Etiology of Acute Kidney Injury: A Clinicopathological Analysis of Phosphate Nephropathy and Review of the Literature

**DOI:** 10.3390/jcm14124081

**Published:** 2025-06-09

**Authors:** Erman Özdemir, Pınar Özdemir, Serap Yadigar, Serkan Feyyaz Yalın, Ergün Parmaksız, Şükran Sarıkaya, Erdoğan Özdemir, Mehmet Rıza Altıparmak

**Affiliations:** 1 Pendik State Hospital, 34890 İstanbul, Turkey; 2 Dr Lütfi Kırdar Kartal Eğitim ve Araştırma Hastanesi, 34865 İstanbul, Turkey; pinarozdemir23@yahoo.com (P.Ö.); serapsert2000@yahoo.com (S.Y.); serkanfyalin@yahoo.com (S.F.Y.); drergnprmksz@hotmail.com (E.P.); sukransarikaya@yahoo.com (Ş.S.); 3 Elazığ Fethi Sekin City Hospital, 23280 Elazığ, Turkey; erdoganozdemir@live.com; 4 Faculty of Medicine, Istanbul University—Cerrahpaşa, 34320 İstanbul, Turkey; mraltiparmak@yahoo.com

**Keywords:** acute kidney injury, biopsy, colonoscopy, sodium phosphate

## Abstract

**Background:** Acute phosphate nephropathy (APN) is an underrecognized cause of acute kidney injury (AKI), typically associated with the use of oral sodium phosphate (OSP)-based bowel preparations. It is characterized by calcium phosphate crystal deposition within the renal tubules and may result in permanent renal impairment. Despite known risks, phosphate-containing solutions are still widely used without sufficient risk stratification. **Methods:** We retrospectively evaluated 517 native kidney biopsies performed in our nephrology clinic between 2017 and 2022. Among these, 12 patients with unexplained AKI and recent colonoscopy history were identified. In nine cases, non-specific tubular deposits on routine staining prompted further histochemical analysis. All had a history of recent OSP-based bowel cleansing. The use of von Kossa staining confirmed calcium phosphate deposition, consistent with APN. **Results:** Out of 517 kidney biopsies performed during the study period, 9 patients were diagnosed with APN based on histopathological findings following recent colonoscopy and OSP-based bowel cleansing. The mean age was 58.7 years, and three were female. Hypertension was present in seven patients, diabetes mellitus in three, and epilepsy in two; one patient had no comorbidities. Baseline renal function was normal (mean serum creatinine 0.86 mg/dL) and increased to 1.76 mg/dL at three months post-exposure. All biopsies revealed tubulointerstitial calcium phosphate deposits and interstitial inflammation; mesangial hypercellularity was observed in five cases, tubular atrophy in three, and acute tubular necrosis in one. All samples stained positive with von Kossa staining. Over time, all patients developed chronic kidney disease, and one progressed to end-stage renal disease requiring dialysis. **Conclusions:** In patients presenting with unexplained AKI and recent OSP-based bowel preparation, APN should be considered in the differential diagnosis. When routine histology is inconclusive, definitive diagnosis may require special histochemical staining. Risk-based restrictions on phosphate-containing agents are warranted to reduce preventable kidney injury.

## 1. Introduction

Phosphate contributes fundamentally to numerous physiological systems, notably cellular energy production, nucleic acid integrity, and skeletal mineralization. The regulation of phosphate balance is achieved through the interplay of gastrointestinal absorption, skeletal storage mechanisms, and renal elimination. Hormonal regulators such as parathyroid hormone (PTH), fibroblast growth factor 23 (FGF23), and 1,25-dihydroxyvitamin D are central to maintaining this equilibrium [1,2]. In situations where phosphate intake exceeds the kidneys’ excretory threshold—such as after ingestion of oral phosphate-based agents (OSP)—transient elevations in serum phosphate can occur. This biochemical disturbance may lead to supersaturation of calcium and phosphate ions, with subsequent precipitation of insoluble calcium phosphate. These crystals preferentially accumulate in distal nephron segments, where slow tubular flow and acidic pH conditions promote deposition. Progressive accumulation may obstruct the tubular lumen, damage epithelial cells, and provoke an inflammatory response. If unresolved, this can result in chronic interstitial fibrosis and persistent loss of nephron mass [2,3]. Acid–base status has a significant influence on calcium and phosphate handling within the kidney. During metabolic acidosis, bone acts as a buffer, releasing stored phosphate and calcium into the circulation. This process increases urinary calcium excretion and raises the likelihood of mineral precipitation within the renal tubules. The distal nephron, in particular, provides a favorable environment for calcium phosphate crystal deposition due to its naturally lower luminal pH. These conditions exacerbate intratubular obstruction and contribute to epithelial injury and inflammation, thereby amplifying the risk of phosphate-induced nephrotoxicity, especially in predisposed individuals [4,5]. Acute phosphate nephropathy (APN) is a clinicopathological entity characterized by acute kidney injury (AKI) that develops days to weeks after the use of OSP-containing bowel preparations or phosphate-based enemas [6]. Although rare, APN remains an underrecognized and underreported cause of renal dysfunction. Its true incidence is uncertain, as few studies have confirmed the diagnosis through histopathological examination [6,7]. In clinical practice, APN is often suspected in patients developing unexplained AKI following OSP exposure, yet the lack of biopsy confirmation has limited precise epidemiological estimates [6]. OSP, an osmotic laxative, has been widely used for colonoscopy bowel cleansing since the early 1990s [8]. In 2008, the U.S. Food and Drug Administration (FDA) issued a class warning underscoring the risk of phosphate-induced kidney injury linked to phosphate-containing bowel preparations [9]. Nevertheless, OSP continues to be widely used in many countries, often without adequate patient risk assessment. The pathophysiology of APN involves transient hyperphosphatemia, intravascular volume depletion, and calcium phosphate crystal precipitation within the distal renal tubules [10,11]. These deposits cause tubular obstruction and injury, leading to interstitial inflammation and potential progression to irreversible renal damage [12,13]. Clinically, APN typically presents with non-specific laboratory findings and bland urinary sediment. Hematuria and pyuria are uncommon, and 24 h urinary protein excretion usually remains below 600 mg, making routine urinalysis largely non-diagnostic [14]. Identified risk factors include older age, female sex, hypertension, diabetes mellitus, extracellular volume depletion, and concurrent use of medications such as angiotensin-converting enzyme inhibitors (ACE-Is), angiotensin receptor blockers (ARBs), diuretics, nonsteroidal anti-inflammatory drugs (NSAIDs), and lithium [6,7,14]. Histologically, APN may present in two phases: an early, reversible form characterized by acute tubular injury or necrosis, and a late, chronic phase showing tubular atrophy and interstitial fibrosis. The chronic form often mimics non-resolving acute tubular necrosis and reflects delayed recognition of the injurious event [12,14]. To date, there is no specific treatment for APN. Hemodialysis may be temporarily beneficial in patients with severe hyperphosphatemia if initiated early, within 24 h of OSP ingestion [14]. However, delayed diagnosis is common, and long-term outcomes are often poor, with many patients progressing to chronic kidney disease (CKD) [6,14]. In light of the continued global use of OSP despite regulatory warnings and the diagnostic challenges surrounding APN, this study aims to raise awareness by presenting one of the most comprehensive biopsy-proven APN series to date. According to our PubMed and Scopus database review, this cohort constitutes the second largest biopsy-confirmed case series of APN in the medical literature. By highlighting the histopathological spectrum and clinical context of these cases, we aim to support early identification of APN and to contribute to efforts that may reduce the future burden of phosphate-associated kidney injury. To further strengthen diagnostic certainty, we retrospectively screened all native kidney biopsies performed in our nephrology unit for unexplained AKI, allowing for a systematic identification of biopsy-proven APN cases linked to OSP exposure.

## 2. Main Points

Oral sodium phosphate and phosphate-containing enemas may cause acute or chronic renal failure.

A renal biopsy is required for a definitive diagnosis of phosphate nephropathy and may be overlooked in routine pathology stains; definitive diagnosis is made with the von Kossa staining method.

Phosphate-free purgatives should be used in preparation for colonoscopy in patients with risk factors.

## 3. Methods

This retrospective observational study was conducted among patients who underwent native kidney biopsy in the nephrology clinic of Kartal Dr. Lütfi Kırdar City Hospital between 2017 and 2022. During this period, a total of 517 native kidney biopsies were performed. Among these, 12 patients were identified with AKI of unknown etiology who had undergone colonoscopy within the preceding six months. In 9 of these biopsies, tubular deposits that could not be clearly classified by routine hematoxylin–eosin (H&E) staining were detected; no such features were observed in the remaining 3 patients. Clinical chart review revealed that all 9 patients had undergone bowel cleansing with oral sodium phosphate (OSP) shortly before the onset of AKI. According to documentation, bowel preparation prior to colonoscopy had been performed using Fleet^®^ Phospho-soda (Prestige Consumer Healthcare; formerly C.B. Fleet Company, Inc., Lynchburg, VA, USA), an OSP formulation available with or without prescription. Each 45 mL dose contains 24.4 g of monobasic sodium phosphate monohydrate and 10.8 g of dibasic sodium phosphate heptahydrate, providing a total of 35.2 g og phosphate per dose. Two such doses, administered approximately 10–12 h apart, correspond to a total phosphate intake of about 70.4 g, equivalent to approximately 20.16 g of elemental phosphorus. Upon identification of this common exposure, additional histochemical analysis with von Kossa staining was performed on the biopsy specimens. The interval between colonoscopy and biopsy was recorded for each patient. Serum creatinine levels obtained at baseline and on days 14, 30, and 90 following colonoscopy were collected. AKI diagnosis was established according to the criteria defined in the Kidney Disease: Improving Global Outcomes (KDIGO) guidelines: an increase in serum creatinine by ≥0.3 mg/dL (≥26.5 µmol/L) within 48 h, or an increase to ≥1.5 times the baseline within seven days, or urine output <0.5 mL/kg/h for six hours [15]. All patients underwent renal ultrasonography to rule out obstructive uropathy. Laboratory assessments included serum creatinine, electrolytes (sodium, potassium, calcium, phosphate), albumin, PTH, and urinalysis. Serological tests to exclude glomerulonephritis included complement levels (C3 and C4), antineutrophil cytoplasmic antibodies (ANCA), proteinase 3 (PR3), myeloperoxidase (MPO), and double-stranded DNA (dsDNA) antibodies. When indicated, serum protein electrophoresis was performed to evaluate for plasma cell dyscrasias. Concomitant use of nephrotoxic agents such as ACE-Is, ARBs, NSAIDs, and herbal supplements was also documented in detail.

All patients were hospitalized and treated with an average of 3000 mL/day isotonic saline intravenously. Kidney biopsy was performed in patients whose renal function did not improve despite adequate volume repletion. Biopsy specimens were processed using standard histological protocols and evaluated by a single renal pathologist who was blinded to the clinical background. Due to the observed tubular deposits, von Kossa staining was applied in addition to conventional staining methods to detect intratubular phosphate deposition. This staining technique is based on a two-step photochemical process in which silver ions bind to tissue calcium salts and, upon exposure to light, are reduced to black metallic silver. The early yellow–brown discoloration reflects the presence of calcium phosphate, followed by darkening due to silver reduction in the organic matrix [16]. Demographic characteristics, comorbidities, biochemical parameters, and pathological findings were comprehensively re-analyzed. Only APN cases confirmed by von Kossa staining and biopsy were included in the final cohort. All confirmed cases were monitored for at least three months for the development of chronic kidney disease, in accordance with the 2024 KDIGO criteria [17]. The study protocol received approval from the Institutional Ethics Committee of Kartal Dr. Lütfi Kırdar City Hospital (approval number: 2022/514/239/6; date: 4 December 2022) and was conducted in accordance with the principles of the Declaration of Helsinki.

## 4. Results

**Figure 1:**
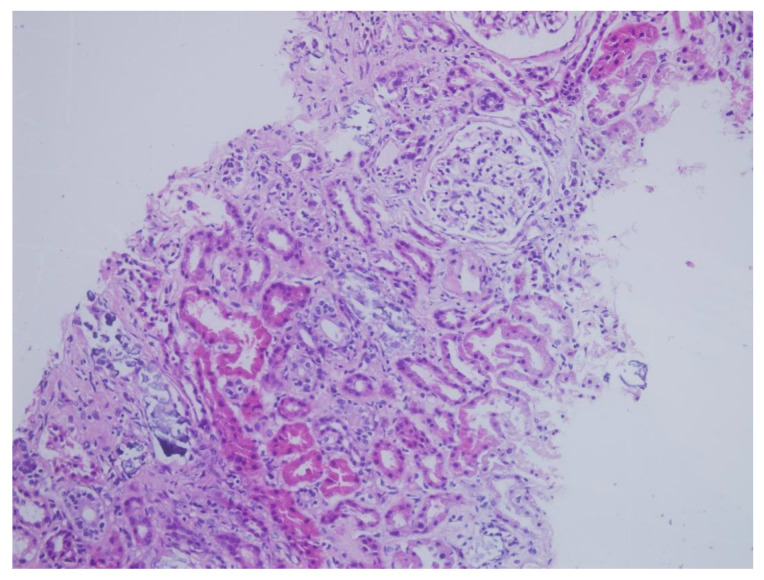
Histological section of renal tissue stained with hematoxylin and eosin (H&E), demonstrating tubular atrophy and interstitial inflammation. Calcium phosphate deposits were not clearly visualized with this stain. Original magnification ×200.

**Figure 2:**
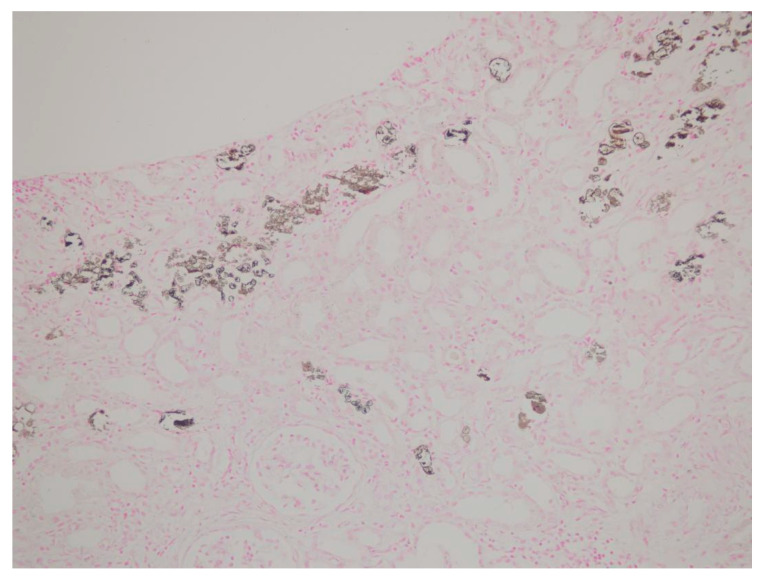
Renal biopsy specimen stained with von Kossa staining, highlighting extensive calcium phosphate crystal deposition within the tubular lumen, confirming the diagnosis of acute phosphate nephropathy. Original magnification ×200.

Between 2017 and 2022, a total of 517 native kidney biopsies were retrospectively reviewed at the nephrology department of Kartal Dr. Lütfi Kırdar City Hospital. Among these, 12 patients were identified who had undergone colonoscopy within the preceding six months and were diagnosed with AKI of unknown etiology. In nine of these biopsies, tubular deposits that could not be clearly classified by routine H&E staining were observed, while the remaining three patients showed no significant pathological findings. These nine cases were identified through a structured screening process based on both histopathological findings and clinical correlation with recent oral sodium phosphate (OSP) exposure. All nine suspicious cases were subsequently evaluated with von Kossa staining, which revealed calcium phosphate deposition in every case. Of the patients definitively diagnosed with APN by biopsy, three were female. The mean age was 58.7 years (range: 48–73 years). Detailed demographic characteristics, comorbidities, current medications, and the interval between colonoscopy and biopsy are summarized in Table 1. Comorbidity analysis revealed diabetes mellitus in three patients, hypertension in seven patients, and a history of epilepsy in two. One patient had no identifiable risk factors. Among the hypertensive patients, six were receiving ACE-Is and/or ARBs, along with diuretics. Two patients were on antiepileptic medications, while one patient was not on any regular medication at the time of diagnosis. Prior to colonoscopy, all patients had normal kidney function (mean serum creatinine: 0.86 mg/dL). However, following OSP exposure, significant elevations in serum creatinine were observed. Mean serum creatinine levels on post-procedure days 14, 30, and 90 were measured as 2.21 mg/dL, 1.87 mg/dL, and 1.76 mg/dL, respectively. These data are presented in Table 1. Urinalysis at admission showed isolated microalbuminuria in two patients (51 mg/g and 78 mg/g), isolated hematuria in one patient (3 erythrocytes/HPF), and isolated leukocyturia in another (21 leukocytes/HPF). One patient exhibited a combination of hematuria, leukocyturia, and microalbuminuria (2 erythrocytes/HPF, 6 leukocytes/HPF, 123 mg/g proteinuria). The remaining patients had unremarkable urinary findings. Renal biopsies were performed at a mean of 67.5 days after colonoscopy. Histopathological evaluation revealed mesangial hypercellularity in five patients, interstitial inflammation in all patients, and mild interstitial fibrosis in two. All biopsies demonstrated tubulointerstitial calcium phosphate crystal deposits. Tubular atrophy was observed in three cases, and one biopsy showed findings suggestive of acute tubular necrosis. No specific findings were observed in any sample with direct immunofluorescence or immunohistochemistry. All specimens demonstrated positive von Kossa staining, confirming phosphate deposition. Histopathological features of the renal biopsies are detailed in Table 2. Representative histological findings from one patient are shown in Figure 1 (H&E staining) and Figure 2 (von Kossa staining). While tubulointerstitial calcium phosphate deposits were not clearly visualized with H&E, they were distinctly revealed with von Kossa staining, confirming the diagnosis of APN. During follow-up, all nine patients diagnosed with APN developed chronic kidney disease. Notably, one patient progressed to end-stage renal disease and required maintenance dialysis. Given that OSP exposure represents a modifiable risk factor, these findings underscore the preventable nature of APN and highlight a significant public health concern. Enhancing awareness among clinicians and pathologists, especially in patients with known risk factors, is critical to preventing iatrogenic kidney injury related to colonoscopy preparation protocols.

Renal biopsies were performed at a mean of 67.5 days following colonoscopy. Histopathological evaluation revealed mesangial hypercellularity in five patients, interstitial inflammation in all patients, and mild interstitial fibrosis in two patients. All biopsies showed tubulointerstitial calcium phosphate crystal deposits. Tubular atrophy was identified in three patients, and one patient had findings consistent with acute tubular necrosis. Direct immunofluorescence and immunohistochemistry studies were negative in all samples. All biopsy specimens demonstrated positive staining with von Kossa, consistent with phosphate deposition. Histopathological findings from renal biopsies are detailed in Table 2. Representative renal histopathological findings from one patient are presented in Figure 1 (hematoxylin and eosin staining) and Figure 2 (von Kossa staining). While tubulointerstitial calcium phosphate deposits were not clearly visualized with hematoxylin and eosin, they were distinctly highlighted by von Kossa staining, confirming the diagnosis of APN.

During follow-up, all nine patients with APN developed CKD. Notably, one patient progressed to end-stage kidney disease, requiring maintenance dialysis. Given that OSP is a modifiable risk factor, these findings underscore the preventable nature of APN and highlight a critical public health concern. Increased awareness among clinicians and pathologists, particularly in patients with underlying risk factors, is essential to prevent iatrogenic kidney injury associated with colonoscopy preparation regimens.

## 5. Discussion

The incidence of APN is not clearly defined. There are no prospective studies on this subject. All available data come from retrospective studies evaluating the reduction in estimated glomerular filtration rate (eGFR) following OSP exposure [6,14]. In this study, we conducted a retrospective analysis to investigate phosphate nephropathy, an often-overlooked etiology of AKI. In this study, the diagnosis of APN was confirmed by correlating recent oral sodium phosphate exposure with histopathological detection of calcium phosphate deposits using von Kossa staining.

Biopsy-proven APN studies published between 2004 and 2022 are summarized in Table 3. A total of 20 publications were reviewed. The three largest studies included 21, 5, and 4 patients, respectively, while the others were individual case reports. Our study is the second largest in terms of biopsy-confirmed APN cases.

The largest case series on APN was a retrospective review of biopsies processed at the Kidney Pathology Laboratory of Columbia University between 2000 and 2004 [18]. Risk factors were evaluated in that study as well. Similar risk factors were observed in our patient population. In one patient, the only identified risk factor was repeated OSP dosing. The remaining patients had multiple risk factors such as diabetes, hypertension, and ACE-I use.

Various medications have been identified as risk factors for APN in multiple studies [6,7,14]. In our cohort, two patients developed APN while receiving antiepileptic treatment. The literature review revealed previously reported cases of kidney injury linked to levetiracetam use. Post-marketing surveillance has also identified instances of interstitial nephritis associated with this drug [36]. These observations suggest that epilepsy or antiepileptic therapy may contribute to the risk of APN, although this association remains unproven. Possible contributing factors include dehydration during the postictal period, reduced fluid intake, and the potential nephrotoxicity of some antiepileptic agents. Further prospective studies are needed to clarify this potential relationship.

In the study by Schaefer et al., an increase in creatinine was observed, on average, 3 to 6 weeks after OSP ingestion, while chronic elevations extended up to 9–15 months [37,38]. In our patients, the time intervals between OSP exposure and the observed rise in serum creatinine were 50, 116, 20, 125, 52, 43, 82, 97, and 28 days, respectively.

The study by Markowitz et alreported that urinalysis findings were often bland, with hematuria and pyuria present only in a few patients. [6,14,18]. Similar findings were observed in our cohort. Studies have indicated that bland urinary sediment and mild proteinuria can support the diagnosis of APN [15]. Our urine analysis results were consistent with the literature.

The most comprehensive pathological study on APN was conducted by Markowitz et al. [18]. The hallmark histological feature of APN is extensive calcium phosphate deposition within the tubular lumen, tubular epithelial cells, and occasionally within the peritubular interstitium. Calcium phosphate crystals can be distinguished from calcium oxalate by positive von Kossa staining and a lack of birefringence under polarized light. Early biopsies typically reveal tubular degenerative changes similar to those seen in acute tubular necrosis (ATN) [18]. In contrast, biopsies performed more than three weeks after OSP exposure usually show chronic changes, including tubular atrophy and interstitial fibrosis. Biopsy findings in our study were consistent with those described by Markowitz et al. [6,14,18]. Tubular necrosis was observed in only one patient whose biopsy was performed within three weeks of OSP exposure. Mild interstitial inflammation was detected in two patients, while chronic changes such as interstitial fibrosis and tubular atrophy were noted in three. Significant ATN was not observed in late biopsies. Six patients showed basement membrane thickening, and five exhibited mild mesangial hypercellularity. Tubulointerstitial calcium phosphate deposits were detected in all nine biopsy specimens. These deposits were confirmed by von Kossa staining, without birefringence under polarized light. The use of von Kossa staining was essential to definitively distinguish calcium phosphate deposits from calcium oxalate.

Notably, one patient progressed to end-stage kidney disease requiring maintenance dialysis. Upon reviewing the clinical history, it was noted that this patient had undergone two separate oral sodium phosphate (OSP) administrations and colonoscopy procedures within a one-month period. The second procedure was performed due to inadequate bowel preparation during the initial colonoscopy. This sequence suggests that repeated exposure to OSP in a short timeframe may carry an elevated risk of phosphate nephropathy. The cumulative phosphate load, combined with insufficient recovery time for renal clearance mechanisms, could have exacerbated tubular injury and crystal deposition. These findings highlight the importance of cautious re-administration of OSP-based regimens, particularly in patients with potential risk factors. It may be prudent to consider alternative bowel preparation strategies or extend the interval between procedures when repeat colonoscopy is necessary. This case underscores the need for heightened clinical awareness and more stringent protocols regarding repeat exposure to phosphate-containing agents. Although the nephrotoxic effects of phosphate-based agents have been known for a long time, this risk is still not sufficiently recognized—even in pediatric patients. In a recent study by Zago et al., it was shown that phosphate enemas were used in children at doses exceeding the recommended levels and that clinical awareness of their toxicity was markedly limited [39]. In the study by Ori et al. [19], female patients predominated, whereas in our cohort, male sex was more common. Hypertension was the leading comorbidity in both studies, highlighting a consistent clinical background despite differences in sex distribution [19].

In the case series by Fernández-Juárez et al. [20], patients were evenly distributed between sexes, while our cohort showed a predominance of male patients. Both studies included individuals within a similar age range and demonstrated comparable baseline renal function, with one patient in each group progressing to end-stage renal disease. Hypertension was a frequent comorbidity across both cohorts, and diabetes mellitus was observed in two and three patients, respectively [20]. Notably, additional risk factors such as epilepsy and hyperlipidemia were present only in our series, suggesting a potentially broader risk profile contributing to the development of APN. The clinical and pathological findings from 17 previously published single-case reports, consolidated in Table 3, demonstrate a high degree of concordance with the results of our study as well as the three larger case series. No significant discrepancies were identified across these datasets [7,12,21,22,23,24,25,26,27,28,29,30,31,32,33,34,35].

## 6. Limitations

This study has certain limitations. Its retrospective design limits causal inference. The relatively small sample size and the absence of a control group reduce the generalizability of the findings. Nonetheless, this study represents the second largest biopsy-confirmed series of APN in the literature, supporting the clinical significance and relevance of its findings.

## 7. Conclusions

OSP usage has been restricted or withdrawn in many developed countries. Unfortunately, it is still commonly used and prescribed in many countries. Physicians performing colonoscopies should be encouraged to utilize phosphate-free bowel preparations. Phosphate nephropathy should always be considered in the differential diagnosis of unexplained kidney injury. A history of colonoscopy must be carefully investigated in such cases. When suspicion arises, pathologists should be alerted to the possibility of phosphate nephropathy, and appropriate histochemical staining should be requested.

## Figures and Tables

**Table 1:** Demographics and renal outcomes in biopsy-confirmed acute phosphate nephropathy cases.

Patient	Age	Sex	Medical History	* Time	Concomitant Medications	Creatinine (mg/dL) ** Day 0	Creatinine (mg/dL) ** Day 14	Creatinine (mg/dL) ** Day 30	Creatinine (mg/dL) ** Day 90
1	57	M	None	50	None	1.14	2.54	1.9	ESRD
2	66	F	DM, HT, HL	116	ACE-Is, statins, OADs	0.8	2.96	2.27	2.4
3	53	F	HT, Asthma, Epilepsy	20	ARBs, CCBs, levetiracetam, ASAs	0.9	1.72	1.63	1.47
4	68	F	DM, Epilepsy	125	Quetiapine, valproic acid	0.8	2.5	1.72	1.68
5	48	M	DM, HT, HL	52	ACE-Is, statins, OADs, ASAs	0.8	1.84	1.7	1.64
6	51	M	HT	43	ARBs	0.7	1.93	1.88	1.71
7	73	M	HT	82	CCBs	0.94	2.19	1.87	1.56
8	59	M	HT, HL	97	ARBs, statins	0.82	1.94	1.86	1.75
9	54	M	HT, HL	28	ACE-Is, CCBs, statins	0.84	2.33	2.04	1.98

M: male, F: female, DM: diabetes mellitus, HT: hypertension, HL: hyperlipidaemia, ACE-Is: angiotensin-converting enzyme inhibitors, CCBs: calcium channel blockers, ARBs: angiotensin receptor blockers, OADs: oral antidiabetic drugs, ASAs: salicylates, statins: * time: duration between colonoscopy and renal biopsy (days), ** day: oral sodium phosphate (OSP) administration (day 0) and the subsequent days of follow-up (days 14, 30, and 90), ESRD: end-stage renal disease.

**Table 2:** Renal biopsy findings in patients with acute phosphate nephropathy.

Parameter	Case 1	Case 2	Case 3	Case 4	Case 5	Case 6	Case 7	Case 8	Case 9
Number of glomeruli	8	5	23	15	14	17	13	13	16
Global glomerulosclerosis	1	1	2	1	1	1	3	4	2
Basement membrane thickening	+	+	+	−	+	−	−	+	+
Mesangial hypercellularity *	Mild	Mild	−	−	Mild	−	−	Mild	Mild
Mesangial expansion *	Mild	−	Mild	Mild	Mild	Mild	−	−	+
Interstitial inflammation *	Mild; Eo, Mn	Mild; Lym	Mild; Mn	Mild; Lym	Mild; Lym	Mild; Lym	Mild; Lym	Mild; Mn	Mild; Lym
Tubular atrophy *	−	Moderate	Mild	−	−	−	Mild	Moderate	−
Tubulointerstitial deposition	+	+	+	+	+	+	+	+	+
Interstitial fibrosis *	−	−	Mild	−	−	−	−	Mild	−
Tubular necrosis	−	−	+	−	−	−	−	−	−
Direct immuno-fluorescence	−	−	−	−	−	−	−	−	−
Immuno-histochemistry	−	−	−	−	−	−	−	−	−
von Kossa staining	+	+	+	+	+	+	+	+	+

Eo: eosinophil, Mn: mononuclear cell, Lym: lymphocyte, * the severity of each lesion was categorized as mild (<25% involvement), moderate (25–50%), or severe (>50%).

**Table 3:** Biopsy-proven acute phosphate nephropathy: literature review of clinical features and risk factors.

Study (Reference)	N	Age/Gender (Min–Max)	Baseline sCr (Min–Max)	* Final sCr (Min–Max)	Medications/Drugs	Co-Morbidities
Markowitz et al. [18]	21	39–82/F = 17, M = 4	0.59–1.60	1.49–3.38 (ESRD = 4)	ACE-Is or ARBs = 14, diuretics = 4, NSAIDs = 3	CKD = 4, DM = 4 HT = 15,
Our study	9	48–73/F = 3, M = 6	0.70–1.14	1.47–2.40 (ESRD = 1)	ACE-Is or ARBs = 5, diuretics = 1, antiepileptic = 2, OADs = 2	Asthma = 1, DM = 3, Epilepsy = 2, HT = 7, HL = 4,
Ori et al. [19]	5	56–73/F = 4, M = 1	0.70–1.19	1.30–3.09	ACE-Is/ARBs = 1	CKD = 3, HT = 5
G. Fernández Juárez et al. [20]	4	61–73/F = 2, M = 2	0.5–1.2	1.7–2.9 (ESRD = 1)	ACE-Is or ARBs = 3, diuretics = 2	DM = 2, HT = 3
Cumulative data from 17 single reports [7,12,21,22,23,24,25,26,27,28,29,30,31,32,33,34,35]	17	50–86/F = 14, M = 3	0.7–1.39	0.85–3.06	ARBs = 7, diuretics = 6, ACE-Is = 3, none = 4, others = 8	DM = 1, Epilepsy = 1, HT = 15, Others = 10
Total	56	39–86/F = 40, M = 16	0.5–1.6	0.85–3.38 (ESRD = 6)	ACE-Is or ARBs = 33, antiepileptic = 3, diuretics = 13, others = 14	DM = 10, Epilepsy =3, HT = 45, Others = 8

Abbreviations: ACE-I, angiotensin-converting enzyme inhibitor; ARB, angiotensin receptor blocker; NSAIDs, non-steroidal anti-inflammatory drugs; OAD, oral antidiabetic drug; HT, hypertension; DM, diabetes mellitus; CKD, chronic kidney disease; HL, hyperlipidemia; ESRD, end-stage renal disease, sCr: serum creatinine (mg/dL), * final sCr: Serum creatinine at ~6 months post-diagnosis (range: 3–12 months).

## Data Availability

The data presented in this study are available on request from the corresponding author.
